# Association between Ideal Cardiovascular Health Metrics and Suboptimal Health Status in Chinese Population

**DOI:** 10.1038/s41598-017-15101-5

**Published:** 2017-11-03

**Authors:** Youxin Wang, Xiaoxue Liu, Jing Qiu, Hao Wang, Di Liu, Zhongyao Zhao, Manshu Song, Qiaofeng Song, Xizhu Wang, Yong Zhou, Wei Wang

**Affiliations:** 1 Beijing Key Laboratory of Clinical Epidemiology, School of Public Health, Capital Medical University, Beijing, 100069 China; 2 Department of Cardiology, Tangshan People’s Hospital, North China University of Science and Technology, Tangshan, 063000 China; 3 Wuhan Hospital of Traditional Chinese Medicine, Wuhan, 430014 China; 4 Beijing Institute of Heart, Lung and Blood Vessel Diseases, Beijing Anzhen Hospital, Capital Medical University, Beijing, 100029 China; 5 School of Medical and Health Sciences, Edith Cowan University, Perth, WA 6027 Australia

## Abstract

Suboptimal health status (SHS) is a physical state between health and illness, and previous studies suggested that SHS is associated with majority components of cardiovascular health metrics defined by American Heart Association (AHA). We investigated the association between SHS and cardiovascular health metrics in a cross-sectional analysis of China suboptimal health cohort study (COACS) consisting of 4313 participants (60.30% women) aged from 18 to 65 years old. The respective prevalence of SHS is 7.10%, 9.18%, 10.04% and 10.62% in the first, second, third and fourth quartiles of ideal cardiovascular health (CVH) metrics (*P* for trend = 0.012). Participants in the largest quartile of ideal CVH metrics show a lower likelihood of having optimal SHS score compared to those in the smallest quartile (odds ratio (OR), 0.43; 95% confidence interval (CI), 0.32–0.59), after adjusting for age, gender, marital status, alcohol consumption, income level and education. Four metrics (smoking, physical inactivity, poor dietary intake and ideal control of blood pressure are significantly correlated with the risk of SHS. The present study suggests that ideal CVH metrics are associated with a lower prevalence of SHS, and the combined evaluation of SHS and CVH metrics allows the risk classification of cardiovascular disease, and thus consequently contributes to the prevention of cardiovascular diseases.

## Introduction

SHS is a physical state between health and disease, and is characterized by the perception of health complaints, general weakness and low energy, and is regarded as a subclinical, reversible stage of chronic health condition^1^. Recently, suboptimal health status (SHS) has become a new public health challenge global wise. SHS shares similar conditions such as unexplained medical syndrome (UMS), chronic fatigue syndrome (CFS), myalgic encephalomyelitis (ME), post-viral fatigue syndrome (PVFS) or chronic fatigue immune dysfunction syndrome (CFIDS)^2^. UMS was reported to be a cause of frequent healthcare usage and account for 20–50% increase in outpatient costs and 30% increase in admission rates in United Kingdom^3^. In China, with the acceleration of rhythms of work and life styles together with the worsening of environmental and pollution, the prevalence of SHS continues to increase, and has been reported that 17.8–60.5% of people currently suffer from SHS^4–6^.

The SHS encompasses 5 characteristics: fatigue, the cardiovascular system, the digestive tract, the immune system and mental status^1^. In a cross-sectional study conducted in workers employed in urban Beijing, we demonstrated that SHS is associated with cardiovascular risk factors and contributes to the development of cardiovascular disease^7^. In a cross-sectional study of Chinese students, SHS was showed to be correlated with lifestyle factors, including physical activity, health responsibility, spiritual growth, interpersonal relations and stress management^8^. Poor work-recreation balance and irregular breakfast eating habits were reported to be associated with increased risk for SHS in a cross-sectional study conducted in southern China^9,10^. In a community-based cross-sectional study conducted in Russia, SHS is showed to be associated with endothelial dysfunction, suggesting integration of suboptimal health status and endothelial dysfunction can be applied to routine screening for risks of cardiovascular diseases^11^. These studies suggest that SHS might contribute to the occurrence of non-communicable chronic diseases (NCD), especially cardiovascular diseases. In order to investigate the causative effect of SHS in NCD, we initiated the China Suboptimal Health Cohort Study (COACS), a longitudinal study starting from 2013^12^. The pilot results of COACS showed that risk factors for chronic diseases such as socioeconomic status, marital status, education, physical activity, salt intake, systolic blood pressure (SBP), diastolic blood pressure (DBP) and total cholesterol (TG) differed significantly between subjects of SHS (SHS score ≥35) and those of ideal health (SHS score <35)^12^.

Taken together, these studies indicated that SHS is associated with majority of cardiovascular health metrics defined by American Heart Association (AHA)^13^. AHA has defined seven behaviors and risk factors (smoking status, body mass index (BMI), physical activity, healthy dietary score, TC, blood pressure and fasting blood glucose (FBG)) as health metrics and created three stages for each metric to reflect poor, intermediate, and ideal cardiovascular health status^13^. A prospective cohort study including 42,847 men in America indicated that majority of cardiovascular events may be preventable through adherence to healthy lifestyle practices^14^ and another study showed that the number of ideal cardiovascular health (CVH) metrics is a strong predictor of cardiovascular disease and mortality^15^.

CVH and SHS are both associated with risk of cardiovascular disease, suggesting that they might be associated or interacted. In this study, we conducted a cross-sectional analysis to explore the potential association between ideal CVH metrics and SHS in Chinese population of COACS from the perspective of preventive, predicative and personalized medicine (PPPM).

## Results

The basic characteristics of participants regarding the scores of ideal CVH metrics are summarized in Table 1. Age, gender, education level, alcohol use, smoking, physical activity, diet, total cholesterol, blood pressure, fasting blood glucose and BMI distribution were different among the four quartile of CVH (*P* for trend <0.001). The prevalence of SHS is statistically different in quartiles of ideal CVH metrics (*P* for trend = 0.012).

**Table 1:** The descriptive characteristics of the participants by quartiles of ideal cardiovascular health metrics.

Characteristic	Ideal Cardiovascular Health Metrics^§^				*P* for tend
	Quartile 1 (n = 1121)	Quartile 2 (n = 797)	Quartile 3 (n = 959)	Quartile 4 (n = 1436)	
Age (years)	37.48 ± 10.85	36.80 ± 10.72	36.05 ± 10.21	37.12 ± 10.31	0.016
Male (%)	869(77.52)	412(51.69)	356(37.12)	357(24.86)	<0.001
Married status(yes)	1008(89.92)	709(88.96)	846(88.22)	1284(89.42)	0.6411
Income, ¥/month^*^					<0.001
≤¥3000	395(35.91)	284(36.41)	288(30.51)	426(29.98)	
¥3001–5000	629(57.18)	431(55.26)	562(59.53)	887(62.42)	
≥¥5001	76(6.91)	65(8.33)	94(9.96)	108(7.60)	
Education level					<0.001
Illiteracy/primary	21(1.87)	16(2.01)	21(2.19)	14(0.97)	
Middle school	352(31.40)	227(28.48)	230(23.98)	373(25.97)	
College/University	748(66.73)	554(69.51)	708(73.83)	1049(73.06)	
Alcohol use					<0.001
Yes	51(4.55)	7(0.88)	11(1.15)	9(0.63)	
No	1070(95.45)	790(99.12)	948(98.85)	1427(99.37)	
Smoking					<0.001
Ideal (never)	436(38.89)	602(75.53)	860(89.68)	1416(98.61)	
Intermediate (former)	11(0.98)	4(0.50)	6(0.63)	2(0.14)	
Poor(current smoker)	674(60.12)	191(23.96)	83(8.65)	18(1.25)	
BMI					<0.001
Ideal(<25 kg/m^2^)	497(44.34)	559(70.14)	754(78.62)	1318(91.78)	
Intermediate(25–29.99 kg/m^2^)	520(46.39)	218(27.35)	201(20.96)	117(8.15)	
Poor (≥30 kg/m^2^)	104(9.28)	20(2.51)	4(0.42)	1(0.07)	
Physical activity					<0.001
Ideal⊕	269(24.00)	264(33.12)	385(40.15)	1136(79.11)	
Intermediate☆	120(10.70)	96(12.05)	118(12.30)	139(9.68)	
Poor (0 min/wk)	732(65.30)	437(54.83)	456(47.55)	161(11.21)	
Diet					<0.001
Ideal (3–4 of components)	109(9.72)	97(12.17)	187(19.50)	615(42.83)	
Intermediate(2 of components)	517(46.12)	466(58.47)	676(70.49)	722(50.28)	
Poor (0–1 of components)	495(44.16)	234(29.36)	96(10.01)	99(6.89)	
Total cholesterol					<0.001
Ideal (<200 mg/dl)	753(67.17)	661(82.94)	846(88.22)	1335(92.97)	
Intermediate (200–239 mg/dl)	368(32.83)	136(17.06)	113(11.78)	101(7.03)	
Blood pressure					<0.001
Ideal (<120/80 mmHg)	294(26.23)	372(46.68)	576(60.06)	1083(75.42)	
Intermediate (SBP129–139 or DBP80–90mmHg)	827(73.77)	425(53.32)	383(39.94)	353(24.58)	
Fasting blood glucose					<0.001
Ideal (<100 mg/dl)	948(84.57)	731(91.72)	891(92.91)	1395(97.14)	
Intermediate (100–125 mg/dl)	173(15.43)	66(8.28)	68(7.09)	41(2.86)	
SHSQ-25 score ≥35	119(10.62)	80(10.04)	88(9.18)	102(7.10)	0.012

^§^Quartile 1, CVH ≤ 9; Quartile 2, CVH = 10–11; Quartile 3, CVH = 12; Quartile 4, CVH = 13–14.

^*^42 subjects provided missing data in variable of income.

^⊕^Defined as ≥150 min/wk moderate intensity or ≥75 min/wk vigorous intensity or ≥150 min/wk moderate + vigorous.

^☆^Defined as ≥1–149 min/wk moderate intensity or1–74 min/wk vigorous intensity or1–149 min/wk moderate + vigorous.

BMI (body mass index), SBP (systolic blood pressure), DBP (diastolic blood pressure), SHSQ-25 (SHS questionnaire 25 items), CVH (cardiovascular health).

Table 2 shows the associations between SHS with the summary score of ideal CVH metrics. Overall, participants who gave higher score of ideal CVH metrics had a lower SHS score (OR, 0.64; 95% CI, 0.49–0.85) when compared those in the largest quartile to those in the smallest quartile of the summary score of CVH), and the association remained statistically significant when age, gender, marital status, alcohol consumption, income level, and education were adjusted (OR, 0.43; 95% CI, 0.32–0.59). In addition, stratified analyses indicated that such negative association is stronger in the male than that in the female, as well as stronger in participants older than 45 years than those younger than 45 years old.

**Table 2:** Associations of suboptimal health status with score of ideal CVH metrics.

Metrics^§^	Total	Sex		Age	
		Men	Women	<45 years	≥45 years
Model 1					
Quartile 1	1.00	1.00	1.00	1.00	1.00
Quartile 2	0.94(0.70–1.27)	0.78(0.51–1.20)	0.85(0.54–1.34)	1.03(0.74–1.43)	0.61(0.29–1.27)
Quartile 3	0.85(0.64–1.14)	0.39(0.22–0.70)	0.81(0.53–1.24)	0.81(0.59–1.13)	0.98(0.52–1.87)
Quartile 4	0.64(0.49–0.85)	0.45(0.26–0.78)	0.50(0.33–0.76)	0.68(0.50–0.93)	0.51(0.28–0.96)
Model 2					
Quartile 1	1.00	1.00	1.00	1.00	1.00
Quartile 2	0.77(0.56–1.05)	0.78(0.50–1.19)	0.83(0.52–1.32)	0.82(0.58–1.16)	0.53(0.25–1.11)
Quartile 3	0.63(0.46–0.85)	0.39(0.22–0.70)	0.79(0.51–1.21)	0.58(0.41–0.82)	0.80(0.42–1.56)
Quartile 4	0.44(0.33–0.60)	0.45(0.26–0.78)	0.49(0.32–0.74)	0.45(0.32–0.63)	0.39(0.21–0.75)
Model 3					
Quartile 1	1.00	1.00	1.00	1.00	1.00
Quartile 2	0.75(0.55–1.03)	0.78(0.50–1.20)	0.78(0.49–1.26)	0.82(0.58–1.17)	0.51(0.24–0.60)
Quartile 3	0.64(0.46–0.87)	0.39(0.22–0.70)	0.80(0.52–1.24)	0.62(0.43–0.89)	0.77(0.40–1.50)
Quartile 4	0.43(0.32–0.59)	0.39(0.22–0.69)	0.50(0.33–0.76)	0.46(0.32–0.66)	0.36(0.18–0.70)

^§^Quartile 1, CVH ≤ 9; Quartile 2, CVH = 10–11; Quartile 3, CVH = 12; Quartile 4, CVH = 13–14.

^*^Model 1: Unadjusted; Model 2: Adjusted for age and sex; Model 3: Adjusted for age (years), sex, marital status, education level, income level.

CVH (cardiovascular health).

The associations between SHS with each CVH metric are listed in Table 3. Never or quit-smoking >12 months, ideal physical activity and ideal dietary intake were significantly associated with the decreased risk of SHS (OR, 0.70 (95% CI, 0.50–0.99), 0.68 (0.54–0.86) and 0.31 (0.22–0.45) for never or quit-smoking >12 months, ideal physical activity and ideal dietary intake, respectively) after adjusting for age, gender, marital status, alcohol use, income level, education level, and other 6 ideal cardiovascular metrics. However, compared to those with intermediate control of blood pressure (SBP 120–139 mmHg or DBP 80–89 mmHg or treated to goal), ideal blood pressures was showed to associate with increased risk of SHS (OR, 1.49; 95% CI, 1.17–1.89) in the full adjusted model. The association between SHS and other CVH metrics (BMI, total cholesterol, fasting blood glucose) were not statistically significant. Stratified analyses showed that the negative correlation of SHS with ideal dietary intake and positive correlation with ideal blood pressure are consistent in men and women and also across different age groups (Table 3).

**Table 3:** Associations of suboptimal health status with each component of cardiovascular health metric, adjusted odds ratio (95% confidence interval)^§^.

Metrics	Total	Gender		Age (years)	
		Male	Female	<45	≥45
Smoking					
Poor	1.00	1.00	1.00	1.00	1.00
Intermediate	1.18(0.26–5.27)	1.20(0.27–5.43)	—	0.93(0.12–7.62)	1.80(0.20–15.94)
Ideal	**0.70(0.50–0.99)**	**0.68(0.47–0.98)**	0.75(0.25–2.29)	0.73(0.50–1.06)	0.67(0.30–1.52)
BMI					
Poor	1.00	1.00	1.00	1.00	1.00
Intermediate	0.76(0.41–1.43)	1.15(0.44–3.00)	0.47(0.20–1.14)	0.72(0.36–1.43)	1.00(0.21–4.77)
Ideal	0.69(0.37–1.28)	0.88(0.34–2.30)	0.48(0.21–1.12)	0.56(0.28–1.10)	1.58(0.35–7.11)
Physical activity					
Poor	1.00	1.00	1.00	1.00	1.00
Intermediate	0.90(0.63–1.27)	0.70(0.40–1.20)	1.04(0.65–1.64)	0.94(0.65–1.37)	0.61(0.23–1.66)
Ideal	**0.68(0.54–0.86)**	**0.50(0.34–0.73)**	0.81(0.60–1.09)	**0.72(0.56–0.94)**	**0.54(0.32–0.91)**
Diet					
Poor	1.00	1.00	1.00	1.00	1.00
Intermediate	**0.65(0.51–0.84)**	0.70(0.48–1.02)	**0.62(0.44–0.87)**	**0.71(0.54–0.94)**	**0.49(0.27–0.89)**
Ideal	**0.31(0.22–0.45)**	**0.35(0.18–0.65)**	**0.29(0.19–0.45)**	**0.34(0.23–0.51)**	**0.27(0.11–0.52)**
Total cholesterol					
Intermediate	1.00	1.00	1.00	1.00	1.00
Ideal	0.98(0.72–1.32)	1.03(0.65–1.63)	0.98(0.65–1.47)	1.07(0.73–1.56)	0.78(0.45–1.32)
Blood pressure					
Intermediate	1.00	1.00	1.00	1.00	1.00
Ideal	**1.49(1.17–1.89)**	**2.00(1.38–2.90)**	1.23(0.90–1.68)	**1.67(1.27–2.20)**	1.01(0.61–1.67)
Fasting blood glucose					
Intermediate	1.00	1.00	1.00	1.00	1.00
Ideal	0.81(0.55–1.20)	0.90(0.49–1.67)	0.76(0.45–1.28)	0.78(0.48–1.27)	0.78(0.40–1.52)

^§^The following potential confounders were adjusted for each OR: gender, age, marital status, alcohol use, income level, education level and the six cardiovascular health metrics.

BMI (body mass index).

## Discussion

To our knowledge, this study is the first attempt to explore the association between SHS and ideal CVH metric score. Subjects in the highest quartile of the ideal CVH metric summary score have a 57% reduced OR of having SHS compared to those in the lowest quartile. We also found that never or quit-smoking >12 months, ideal physical activity and ideal dietary intake are independently protective factor of SHS, while ideal control of blood pressure is risk factor of SHS.

The main findings in the present study are that ideal CVH metric score is negatively correlates with SHS score in a large population. This association is independent of the known confounding factors, i.e., age, gender, education level, married and alcohol drinking. It suggests that increasing ideal CVH metric score is a new independent protection factor of SHS (besides the already existing list of factors, such as age, gender, marital status, alcohol consumption, income level, and education), and can be applied as parameters to further address the biological characteristics of SHS. Moreover, it can be proposed that intervention on SHS together with maintaining ideal CVH may be a joint effective way for the prevention of cardiovascular disease from the perspective of PPPM.

Consistent with our previous study^7^ and other studies which reported that unhealthy lifestyle determined by the Health-promoting Lifestyle Profile (HPLP-II), work-recreation balance and breakfast eating habits were also reported to be associated with SHS in multiple studies^8–10,16^, healthy diet intake is associated with a lower risk of suffering SHS, and smoking or physical inactivity are associated with risk of SHS. Taken together, health behaviors play important roles in contributing to the association between SHS and risk of cardiovascular events.

The present finding that intermediate control of blood pressure negatively correlates with SHS score is inconsistent with our previous study that blood pressure is elevated in the participants with higher SHS score. A cross-sectional study conducted in Samara of Russia showed that blood pressures are not statistically correlated with SHS score (beta = 0.069, p = 0.199 and beta = −0.040, p = 0.416 for SBP and DBP, respectively)^10^. These inconsistencies remain unexplained, but there are 3 possible explanations. Firstly, internal correlation among CVH and confounding factor may lead to the inconsistent association. For example, high levels of physical activity and current smoking status had negative relations to overweight or obesity^17^, and there was report that systolic blood pressure markedly declined from non-smokers to smokers^18^. Secondly, the subjects of hypertension had been excluded from the recruitment, might leading to that the measures of blood pressure and triglycerides were slightly lower in subjects of SHS than those of health^12^. Finally, abnormalities of blood pressure variability might be characteristic of SHS. In concept, SHS covers confoundedly the symptoms of CFS, which is a medical condition characterized by long-term fatigue and other symptoms that limit a person’s ability to carry out ordinary daily activities^19^. Abnormalities of blood pressure variability occurs in CFS^20,21^, and even lower blood pressure and abnormal diurnal blood pressure regulation occur in patients with CFS^18^. Summarily, the inconsistent association between SHS and blood pressure remains unexplained, which need to be further validated in large sample-size cross-sectional or longitudinal designed studies.

Although our study includes a large sample size and adjustment for a variety of potential confounders, several limitations should be noted. Firstly, the design of cross-sectional study makes it hard to infer the causal-effect relationship between SHS and CVH metrics, which need be validated in prospective cohort studies or clinical trials. Secondly, dietary intake was defined based on salt intake instead of an established food frequency questionnaire, and thus may result bias. Secondly, all participants in this study were from the COCAS in *Jidong* community of mining industry with relatively high income status and attained higher education when compared to the general Chinese population. Therefore, our results are limited in generalization to the general population in China. Finally, we did not collect sufficient information on the pre- or post-menopause status of women, which appears to be a potential factor of SHS in women.

## Conclusions

Higher ideal CVH metrics are associated with a lower prevalence of SHS. The evaluation of SHS combined with the analysis of CVH metrics allows the risk classification of cardiovascular disease, which may consequently contribute to the prevention of cardiovascular diseases.

## Methods

### Ethics Statement

The study was conducted according to the guidelines of Helsinki Declaration. Ethical approvals were obtained from the Ethics Committee of Capital Medical University. Written informed consents were obtained from all participants.

### Participants

All 4313 participants (60.30% women) aged range from 18 to 65 years old were recruited from COACS^12^. Including and excluding criteria can be referred to our previous publication^12^. Briefly, we included all adults (from 18 to 64 years old) participated in the baseline investigation and excluded these currently suffering from diabetes, hypertension, self-reported hyperlipemia, cardiovascular or cerebrovascular conditions (including self-reported atrial fibrillation, atrialflutter, heart-failure, myocardial infarction, transient ischaemic attack, and stroke), any type of cancer and gout. From 2013 to 2014, all participants underwent a standardized physical examination, including medical history, anthrophysical measures, blood hematology and biochemistry analysis, rest electrocardiography, abdominal ultrasonography, and all participants were asked to complete the SHSQ-25 under the instructions of well-trained researchers.

### Determination of SHS

The SHS score was measured using SHS questionnaire 25 items (SHSQ-25), a self-reported survey tool validated in various populations^2,7,11,22,23^. SHSQ-25 consists of 25 items of 5 domains of 1) fatigue, 2) the cardiovascular system, 3) the digestive tract, 4) the immune system and 5) mental status. A score ≥35 represents a SHS and <35 represent an ideal health^1,12,23^.

### Assessment of Cardiovascular Health Metrics

According to the guidelines by America Heart Association, we defined the 7 CVH metrics at 3 levels: “ideal”, “intermediate” and “poor”^13^. Data on smoking, physical activity and dietary intake were collected via questionnaire^12^. Smoking metric was classified as ideal (never or quit-smoking >12 months); intermediate (former-smoking ≤12 months) or poor (current smoking). Physical activity was classified as ideal (≥150 min/week of moderate intensity or ≥75 min/week of vigorous intensity), intermediate (1–149 min/week of moderate intensity or 1–74 min/week of vigorous intensity), or poor (none). Referred to our previous literatures^24,25^, dietary intake, mainly based on salt consumption, were graded into ideal (<6 g per day), intermediate (6–10 g per day) or poor (>10 g per day). BMI was classified as ideal (<25 kg/m^2^), intermediate (25–29.9 kg/m^2^) or poor (≥30 kg/m^2^). Blood pressure was classified as ideal (SBP <120 mmHg and DBP < 80 mmHg and untreated), intermediate (SBP 120–139 mmHg or DBP 80–89 mmHg or treated to goal), or poor (SBP ≥ 140 mmHg or DBP ≥ 90 mmHg). FBG was classified as ideal (<100 mg/dL and untreated), intermediate (100–125 mg/dL or treated to goal), or poor (≥126 mg/dL). TC was classified as ideal (<200 mg/dL and untreated), intermediate (200 to 239 mg/dL or treated to goal), or poor (≥240 mg/dL).

### Statistical Analyses

Normality distributions of continuous variables were tested by the Kolmogorov Smirnov tests. Continuous variables underlying normal distribution were described with mean together with standard deviation (SD) and compared using ANOVA, otherwise using nonparametric methods. Categorical variables were described with percentages and compared using Chi-square test. Logistic regression was used to analyze the association between each CVH metric and presence of SHS, and the result was presented using the odds ratios (ORs) and 95% confidence interval (CI), adjusted for age, gender, marital status, alcohol use, income level, and education level in the models.

To assess the association between ideal CVH metrics with SHS, we calculated a summary score of ideal CVH metrics. Each CVH metric was assigned a score as follows: “poor” was coded as “0”, the “intermediate” was coded as “1”, and “ideal” was coded as “2”. The summary score of ideal CVH metrics for each individual was the sum of the scores of his/her 7 CVH metrics. In the logistic regression models estimating the association between the summary score of CVH and SHS, the summary score was entered in the models as quartiles (with the lowest quartile as the reference). In addition, we estimated the association between CVH metrics and SHS stratified by gender and age groups.

All statistical tests were 2-sided with the significant *P* level set at 0.05. All analyses were performed using SAS 9.4 (SAS Institute, Cary, North Carolina, USA).

### Data availability statement

All relevant data are published within the paper and its supporting additional files.
